# Attenuated brain activity during error processing and punishment anticipation in procrastination – a monetary Go/No-go fMRI study

**DOI:** 10.1038/s41598-019-48008-4

**Published:** 2019-08-07

**Authors:** Marek Wypych, Jarosław M. Michałowski, Dawid Droździel, Magda Borczykowska, Michał Szczepanik, Artur Marchewka

**Affiliations:** 1 Laboratory of Brain Imaging, Nencki Institute of Experimental Biology of Polish Academy of Sciences, Warsaw, Poland; 2 Institute of Psychology, SWPS University of Social Sciences and Humanities, Poznań, Poland; 3 Faculty of Psychology, University of Warsaw, Warsaw, Poland

**Keywords:** Human behaviour, Motivation

## Abstract

Procrastination is a self-regulatory failure in which people voluntarily but irrationally delay important tasks. Trait procrastination is estimated to affect 15–20% of the total population and leads to a significant decrease in performance, satisfaction with achievements, and quality of life. Procrastination is related to impulsivity and reduced executive control, especially in the domain of inhibition. Moreover, procrastinatory tendencies seem to increase with negative affect, suggesting impaired emotion regulation. The aim of this study was to investigate the neuronal mechanisms of inhibition, error processing, and behavioral control under pressure of punishment in procrastinators. Non-student subjects recruited to low (LP) and high procrastination (HP) groups performed an fMRI monetary Go/No-go task. HP showed significantly lower error-related activity in ACC than LP. There was also a significant group by condition interaction in the ACC and right DLPFC suggesting increase of control during the punishment condition in LP but not HP group. These results suggest that procrastinators have impaired error processing mechanisms which may add to the persistence of procrastination through difficulties in correction of faulty behaviors. Procrastination also seems to be related to a decreased ability to intensify self-control in more demanding situations and/or impaired coping in the context of negative situations.

## Introduction

Procrastination is a self-regulatory failure in which people voluntarily but irrationally delay some tasks, despite knowing that such a behavior will lead to discomfort. This may refer to job-related as well as everyday tasks (e.g. preparing a yearly report on New Year’s Eve, writing conference abstracts at the deadline, filling income tax return forms after the deadline, leaving Christmas shopping until Christmas Eve, postponing learning until the last night before the exam, etc.). The problem of trait procrastination is very common - it is estimated to affect 15–20% of the total population^1,2^ and 50–95% of students^3^ (see^4^ for review). Postponing tasks leads to a significant decrease in performance^5^ and affects satisfaction obtained from achievements as well as quality of life^4^. Negative effects of procrastination are also noticed in the field of economics^6,7^ and in health care due to delayed seeking of treatment^8,9^. Thus, the problem of procrastination is gaining more and more attention among researchers representing different fields of science and different forms of interventions are being tried (for metaanalyses see^10,11^).

Researchers attempt to explain procrastination by looking at the phenomenon from many different perspectives: personality traits^12,13^ (for review see^4^), decision-making style^14^, time perspective^15,16^, temporal motivation^4^, temporal discounting^17^, emotion regulation difficulties^18,19^, executive dysfunctions^20–22^ etc. Recently researches have started to work towards elucidating the biological correlates and basis of procrastination using both behavioral genetics^21,23,24^ and brain research^17,25–27^. All of these approaches have resulted in valuable contributions to our understanding of procrastination while, at the same time, demonstrating the complexity of this phenomenon.

Previous studies revealed strong relationships between procrastination and impulsivity (r = 0.41) as well as other impulsivity-related traits such as lower conscientiousness (r = −0.62) and weaker self-control (r = −0.58; metaanalysis in^4^). Impulsivity seems to be particularly important because it is thought to share biological background with procrastination both on genetic^21,23^ and neuroanatomic^25^ levels. Steel^28^ even suggested that procrastination might be an “evolutionary byproduct of impulsivity”. Relationship between impulsivity and procrastination may explain procrastinators’ difficulties in executive function that have been found in several studies. Self-reported executive dysfunctions were found to be a strong predictor of procrastination^20^. On the behavioral level, procrastination has been found to be correlated with executive dysfunctions in the domain of inhibition in anti-saccade, Stroop, and stop-signal tasks^21^. Interestingly, no relation between procrastination and stop-signal reaction time in the stop-signal task was found by Rebetez and colleagures^22^. In the same study, however, a relationship between procrastination and poorer inhibition was revealed in a different, more complex task measuring resistance to proactive interference in working memory, however only in women with higher level of negative affect. Importantly negative emotions, including task aversiveness, feelings of stress, anxiety, or guilt, seem to intensify procrastinating behaviors^18,19^ which, through a vicious circle, leads to exacerbation of stress, guilt, and/or anxiety. Emotional regulation difficulties where also shown to be related to procrastination^18,29^ and emotion regulation training focusing on building skills to tolerate and modify aversive emotions has been shown to reduce procrastination^30^. Poor emotion regulation and/or ineffective stress coping seems to be one of the crucial factors in procrastination.

Impulsivity, deficits in inhibition, and emotion regulation difficulties are commonly found in substance use and behavioral addictions (exemplary metaanalyses in^31–33^ respectively). To our knowledge, there is only one conference proceeding looking at the comorbidity of procrastination and addictions^34^. The authors showed that within psychotherapy patients, those who met their diagnostic criteria for procrastination got significantly higher results in substance use. This may suggest that procrastination and addictions could share some common underlying mechanisms. It has already been postulated that ineffective emotion regulation and poor impulse control undermine self-control in general and could underlie procrastination, addictions and other self-control disorders^35^.

Steel suggested procrastination to be an effect of the interplay between the limbic system and prefrontal cortex (PFC)^28^. Around the same time, in their studies of executive dysfunctions in procrastinators, Rabin and colleagues^20^ proposed the use of neurobiological tools in research of procrastination. However, to the best of our knowledge, there are only few published studies addressing potential neuronal correlates of procrastination: three employed resting-state fMRI^26,27,36^, one task-related EEG^17^, and two used MRI to look into neuroanatomical correlates of procrastination^25,37^. Most of the results of these studies can be interpreted as pointing at impulsivity-related patterns of brain activity and structure in procrastination. They also suggest that procrastinators exhibit lower capability to make decisions favoring long-term goals and difficulties in inhibition of undesired behaviors. However, to our knowledge, there are no task-fMRI studies published concerning procrastination.

The aim of this study was to investigate the neuronal mechanisms of inhibitory control and error processing in high procrastinators (HP) as compared to a low procrastination (LP) control group. Additionally, we sought to examine the influence of context inducing negative emotions (in this case, risk of financial punishment) on the neural mechanisms of control in procrastinators. Our previous behavioral study employing a monetary Go/No-go task showed difference in error processing (i.e. post error slowing) between low and high procrastinators in the punishment condition^38^. In the present study we applied a monetary Go/No-go task in the mixed block/event-related design inside the MRI scanner^39^. The Go/No-go task was performed in three different conditions: Neutral (NEU) – where monetary gratification did not depend on performance; Reward (REW) – where each correct response to the Go signal and each correct inhibition of No-go trials were rewarded with small amounts of money; and Punishment (PUN) – where subjects were given some money prior to the task and each error resulted in a loss of a fraction of the money.

For practical reasons, most of procrastination research involves student subjects. However, we have shown that students and non-students (or young adults and adults) can differ in several procrastination-related aspects^29^. Those differences could result from differences in maturation of the prefrontal cortex as its myelination is often not complete at the age of a typical student^40^. To avoid this issue we decided to invite non-student subjects (aged over 25) to participate in the current study.

Taking into account procrastinators’ deficits in inhibition^21,22^ one might expect lower brain activity during inhibition, similar to what has been found in studies on impulsivity^41,42^ and substance dependence (review in^43^). On the other hand, in behavioral addictions (gambling and binge eating, review in^43^; or internet gaming disorder^44,45^) the opposite has been found – increased inhibitory brain activity which has been interpreted as the compensatory mechanism. Thus we wanted to check if and in which direction differences could be found in the activities of inhibition related brain areas (i.e.: inferior and middle frontal gyri, anterior cingulate cortex (ACC), insulae, parietal lobules, putamens; reviews in^46,47^ during inhibition trials between high and low procrastinator groups.

Procrastination is a persistent behavior and procrastinators often fail to correct it despite their honest intentions. We hypothesize that impairments of error processing mechanisms underlie this difficulty. We expect lower brain activity after errors, similar to that found i.a. in substance (review in^43^) and/or behavioral addictions (review in^43^, see also^45^), or more generally within regions typically engaged in error processing. These areas include the ACC, presupplementary motor area (pre-SMA), inferior and middle frontal gyri, insulae, precunei, and inferior parietal lobules (metaanalysis in^48^). On the behavioral level, we expected shorter/lack of post-error slowing in procrastinators, especially in the punishment condition (cf. student population^38^).

Effective coping in more demanding situations (e.g. related to emotional load) require intensified self-control. Taking advantage of the mixed block/event-related design applied in the current study we expect to observe the influence of negative context on the “tonic” (or sustained) i.e. context- or block-related brain activation during entire punishment blocks. Such punishment-related changes in tonic brain activity has already been shown in a very similar study on the general population. Stronger, sustained activity of ACC during the punishment blocks was interpreted as intensified monitoring aimed at preventing possible losses^39^. Negative affect however seems to undermine self-control in procrastinators^18,35^. Thus we hypothesize that we will find a significant interaction between the group (LP > HP) and emotional context of the task (PUN > NEU) in the activation of prefrontal “control” brain regions. Specifically, we believe there will be differences in the activity of the right dorsolateral prefrontal cortex (DLPFC) which is known to be crucial for behavioral control. Moreover, right DLPFC showed higher activity in tasks requiring integration of cognitive and emotional processes (e.g.^49^; review in^50^). These differences could also be expected in areas showing stronger activity during punishment in a similar experiment in a standard population, i.a.: medial, middle and inferior frontal gyri, cingulate, pre-SMA, caudates and insulae, cf.^39^. The reward condition was added for exploratory analyses in similar areas with no particular directional hypotheses.

## Methods

The protocol of the study was approved by the Ethics Committee of the Faculty of Psychology at the University of Warsaw in accordance with the Declaration of Helsinki.

### Subjects and procedure

Subjects were recruited from the non-student population (aged 25–45) based on the Pure Procrastination Scale (PPS; see further “Questionnaires” section). Information about the study and a request to complete the electronic version of the PPS questionnaire was spread via social media and an e-mail list of the Nencki Institute. Polish version of PPS has not been normalized on representative sample, and most questionnaires norms may be affected by culture and the differences in translation^51^. Thus we decided to use the within-group norms based on subjects who took part in the recruitment process rather than values reported by the author of PPS^52^. Indeed, in our sample the mean (converted to 5-point scale) was significantly lower (2.79 + /− 0.96; N = 204) than reported by Steel (3.42 + /− 0.85; N = 4169)^52^ (p < 0.0001). Out of 204 subjects who completed the PPS, 40 right-handed individuals were invited to take part in the fMRI study. Subjects with scores below 1 SD from the mean (PPS ≤ 20) were recruited to the low procrastination (LP) group and those scoring above 1 SD from the mean (PPS ≥ 43) to the high procrastination (HP) group. Four subjects were excluded from final analysis (one subject fell asleep in the MRI scanner, one neglected to disclose their antidepressant drug therapy prior to scanning, one subject didn’t respond correctly to the task, and data of one subject had to be excluded due to technical problems with response-pad during the task). Data from 36 subjects (see Table 1) are analyzed in this study.

**Table 1:** Demographic and psychometric group characteristics.

	Low Procrastination (LP)	High Procrastination (HP)
N	18 (10 ♀)	18 (8 ♀)
age	32.8 ± 5 (range: 26–42)	31.9 ± 4.6 (range: 25–41)
PPS score	18.7 ± 2.9 (range: 13–20)	49.3 ± 4.8 (range: 43–59)

Participants were first asked to complete the MRI-safety questionnaire, then they were informed about the details of the study, and asked to go through a short training session in the 0 T mock-scanner. The training consisted of three blocks, presenting neutral (NEU), punishment (PUN), and reward (REW) conditions. Except for the fact that training blocks lasted for 30 sec and no real money was involved, the training was identical to the proper experiment (see task description for details). At the end of the training session, participants’ averaged Go reaction time (RT) was displayed on the screen. Subjects were informed by the experimenter that they would have 50 ms more than the displayed result, so if they could keep their RT, most of Go trials should be considered correct. In fact, during the fMRI experiment the threshold for counting monetary gratification was constant and set to 500 ms for all participants.

After the training, two piles of money were put on the table in front of participants: 48 PLN guaranteed for participation in NEU blocks (8 PLN for each block), and 60 PLN from which they would lose for incorrect responses in the PUN blocks. The empty place on the table next to the two piles of money was described as a 0 PLN pile that will grow with each correct response in the REW blocks with maximum possible gain of 60 PLN (see task description below). After that, subjects were asked to sign informed consent and were invited to the MRI scanner.

### Go/No-Go task

**Figure 1:**
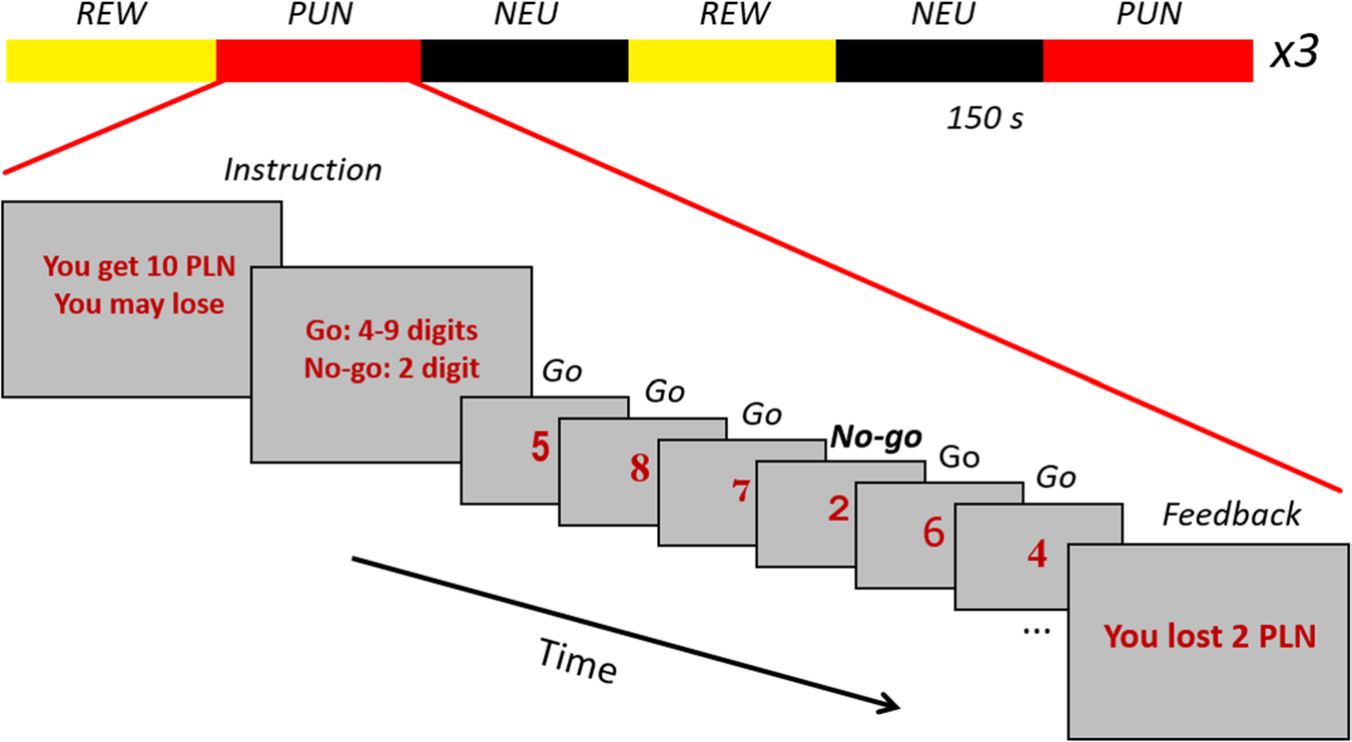
Experimental design of monetary Go/No-go task. Digits displayed in randomly changing fonts for 250 ms were used as Go and No-go signals with a randomized inter-trial interval between 1 and 1.5 sec. The task consisted of three types of blocks: neutral (NEU), reward (REW), and punishment (PUN). Each block lasted for 150 sec and consisted of 100 Go and 20 No-go trials. In the NEU condition, monetary gratification (8 PLN per block) did not depend on the subjects’ performance. In REW each correct response to the Go signal and correct inhibition for No-go trials were rewarded with small amounts of money (0.05 and 0.25 PLN respectively), while in the PUN condition subjects were given 10 PLN prior to the block and each error resulted in a loss (0.05 PLN for either slow or lack of response to the Go signal and 0.25 PLN for a response to the No-go signal).

The Go/No-go task^39,53^ was implemented in Presentation software (Neurobehavioral Systems, Inc., Berkeley, CA, www.neurobs.com). The participants’ task was to press a response-pad button as fast as possible when one of the Go signals (digits 4–9) appeared on the monitor and refrain from pressing the button after the occurrence of No-go signals. Digits 1, 2, and 3 were used as No-go signals, but in each block only one of the digits was used (see Fig. 1).

Digits were displayed for 250 ms in randomly changing fonts. The inter-trial interval was randomized and varied between 1 and 1.5 sec. The distance between No-go trials was at least 4 Go trials, to allow event-related analysis of BOLD signal related to No-go trials, however sometimes (one time in half of the blocks) two No-go trials in a row were displayed to prevent subjects from reaching the conviction that after a No-go trial there is always a Go trial.

The task consisted of three different conditions: neutral (NEU), reward (REW) and punishment (PUN). In the NEU condition monetary gratification (8 PLN or ~2 USD in each block) did not depend on the subjects’ performance. In REW each correct response to the Go signal and correct inhibition for No-go trials were rewarded with small amounts of money (0.05 and 0.25 PLN respectively) with a maximum possible gain of 10 PLN in one block. In the PUN condition, subjects were given 10 PLN prior to the block and each error resulted in a loss of a fraction of the money (0.05 PLN for a slow response or lack of a response to the Go signal and 0.25 PLN for a response to a No-go signal). Each block lasted 150 sec and consisted of 100 Go and 20 No-go trials. There were three consecutive fMRI sessions, each consisting of 6 blocks (two of each condition in every session). The order of blocks was pseudorandom (see Fig. 1), different in each session but identical for all the subjects from both groups.

Instructions and information about block type was displayed on the screen before each block. Additionally, block type was coded with the color of the displayed digits (NEU - black, REW- golden-yellow, and PUN - red). After each of the blocks, participants were informed about the monetary gratification (in NEU and REW) or monetary loss (in PUN condition). To automatically calculate the income/loss, the response to the Go signal was considered correct if it was faster than 500 ms. After the experiment, participants were paid according to their performance (146.69+/− 10.14 PLN; ~35 USD).

### Questionnaires

Subjects were recruited based on their results in the Pure Procrastination Scale (PPS)^52^ in its Polish adaptation^54^. PPS in currently one of the most widely used scales in procrastination research. It is a 12-item measure constructed from items chosen from three other measures of procrastination: Adult Inventory of Procrastination^55^, Decisional Procrastination Questionnaire^56^, and General Procrastination Scale^57^. PPS showed higher correlation with the validation scales (Satisfaction with Life Scale, Susceptibility to Temptation Scale and Irrational Procrastination Scale) than the full questionnaires it was based on^52^ and shows good reliability with Cronbach’s alpha, ranging between 0.89 and 0.93 depending on the adaptation^58^.

After MRI scanning, subjects were asked to fill out the Polish version^59^ of the UPPS-P impulsivity questionnaire^60^ and the short form^61^ of Sensitivity to Punishment and Sensitivity to Reward Questionnaire (SPSRQ-SF)^62^, in its Polish version^63^. The latter questionnaire measures subject’s proneness to seek rewards and to avoid punishments.

### Behavioral data analysis

The Go trial reaction times (RT), number of errors of commission to No-go trials, and Post Error Slowing (PES), defined as the mean difference between RTs in Go trials after incorrect and correct responses to preceding No-go stimuli, were analyzed using repeated-measures ANOVA using SPSS 21 software (IBM). Greenhouse-Geisser corrections for the number of degrees of freedom were applied if the test assumptions were violated. Go trial responses faster than 200 ms were considered as guessing and excluded from further analysis (0.72% of all trials). All of the other responses to Go signals (including those slower than 500 ms) were included in computation of RTs.

### fMRI data acquisition and preprocessing

MRI data were collected on a Siemens 3 T Trio MRI scanner using a 32-channel coil. High resolution (1 × 1 × 1 mm voxels) T1 weighted anatomical images were acquired with the following parameters: TR = 2530 ms, TE = 3.32 ms, flip angle = 7°, 176 1 mm thick slices, FOV = 256 × 256 mm. Functional images (3 × 3 × 3 mm voxels) were acquired using an echo planar imaging pulse sequence with parameters: TR = 2000 ms, TE = 30 ms, flip angle = 90°, 36 interleaved 3 mm thick slices, FOV = 216 × 216 mm. Additionally, a field-map was acquired to allow for field inhomogeneity distortion correction.

MRI data were preprocessed and analyzed in SPM12b (http://www.fil.ion.ucl.ac.uk/spm/software/spm12/). Data from each subject underwent the same preprocessing steps (motion correction and unwarping, co-registration of the T1 image to mean EPI image and segmentation of the T1 image to different tissues). A DARTEL^64^ template was created based on gray and white matter images of all subjects, fMRI data were normalized to MNI space with 2 × 2 × 2 mm voxels, and smoothed with a 6 mm Gaussian kernel.

### fMRI data analysis

Normalized, smoothed images were then used to compute 1^st^ level General Linear Model (GLMs) separately for each subject. For each subject the 1^st^ level GLM consisted of three sessions, each containing nine regressors of interest. On the event-related level, only No-go trials were included in the model which therefore contained six event regressors: separately, correct and incorrect No-go trials for each (NEU, REW, PUN) condition. Additionally, three block regressors indicating periods of NEU, REW and PUN blocks were put in the same model to account for the task contexts. Including event and block regressors in a single model makes it easier to interpret the results as event related brain activity during inhibition (correct No-go trials) and error-processing (incorrect No-go trials) can be separated from the tonic context related brain activity (lasting along whole NEU, REW and PUN blocks; cf.^39^). Prior-block instructions, after-block feedbacks, double-No-go trials (occurring a single time in half of the blocks) were entered into the GLM as covariates of no interest. All the regressors related to the course of the experiment were convolved with standard haemodynamic response function implemented in SPM12b software. Additionally, 6 head-motion parameters, as well as regressors representing each of three scanning sessions, were entered into the GLM as covariates of no interest. As each of the NEU, REW and PUN blocks lasted for 150 sec the expected block-related signal changes had a period of ~300 sec. In order not to filter those possible changes out, the high-pass filter cutoff was set to 650 sec, a value exceeding four lengths of the single block (including inter-block intervals) to allow for the between block comparisons.

Several 2^nd^ level (or group) analysis were performed. Brain activity during correct inhibition was analyzed based on event-related 1^st^ level analysis (correct No-go trials in NEU condition) using one-sample (HP or LP) and two-sample (HP vs LP) t-tests. Error processing brain activity (erroneous responses to No-go trials in NEU condition) were analyzed the same way. The influence of context on tonic brain activity was tested in the interaction analysis using the flexible-factorial models based on the 1^st^ level block results. Additional flexible-factorial models were prepared to analyze possible group x condition interaction in error processing (based on event related 1^st^ level contrasts from erroneous responses to No-go trials in different conditions).

In the two-sample t-tests and the interaction analyses, small-volume corrections (SVC) were applied to clusters found in areas described in the hypothesis if the clusters exceeded an arbitrary threshold of 20 voxels at p = 0.001, uncorrected. The significance was then tested in a spherical small volume of 16 mm radius (about 2145 voxels). In the one-sample t-tests (presented in the supplementary materials) cluster size correction for family-wise error was used to account for multiple comparisons.

BSPMVIEW toolbox v.20161108^65^ was used to generate tables of activated regions. All peaks of activation were labeled using the Harvard-Oxford atlas (https://fsl.fmrib.ox.ac.uk/fsl/fslwiki/Atlases). Thresholded statistical maps were plotted using Nilearn v.0.4.0 (http://nilearn.github.io/).

## Results

### Behavioral results

**Figure 2:**
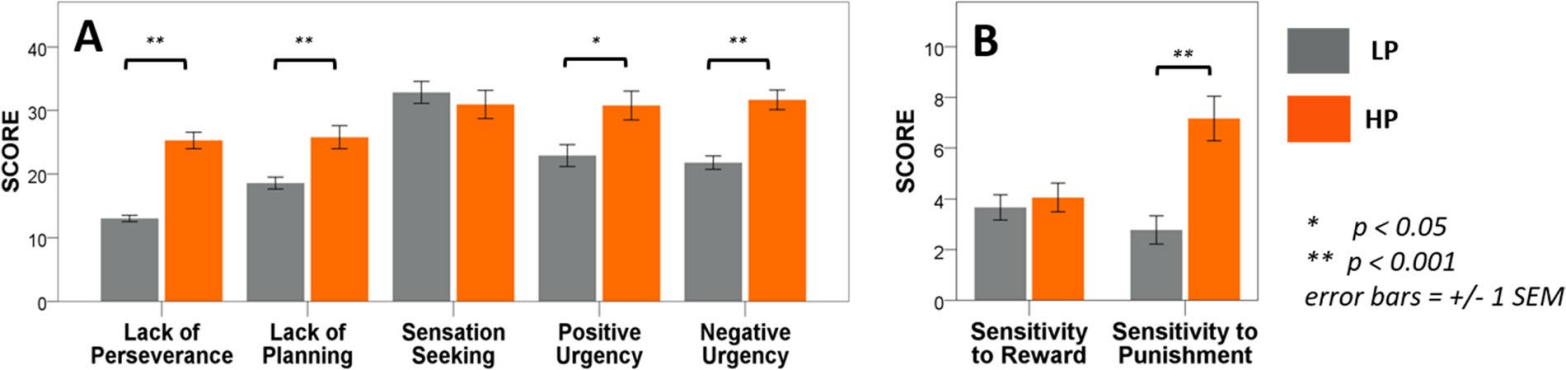
Questionnaire results. High procrastinators (HP, orange) scored significantly higher in 4 scales of the UPPS-P impulsivity questionnaires (**A**) and in the Sensitivity to Punishment scale of the SPSRQ-SF (**B**).

Procrastinators are significantly more impulsive – they scored significantly more on 4 out of 5 scales of UPPS-P: lack of perseverance, lack of planning, negative urgency and positive urgency (ts > 2.77, ps < 0.01). No difference was found in sensation seeking (t = 0.67, p = 0.51; Fig. 2A).

No significant difference was found in the sensitivity to reward scale of SPSRQ-SF, t = 0.52, p = 0.61, but procrastinators are much more sensitive to punishment, t = 4.22, p < 0.001 (Fig. 2B).

**Figure 3:**
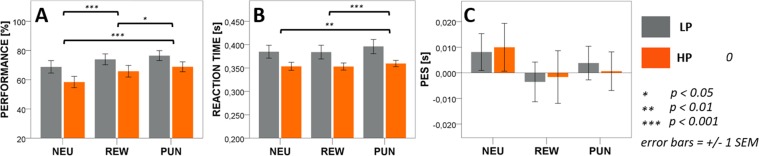
Go/No-go task behavioral results. No significant between group differences were found in task performance (**A**), reaction times (**B**) nor in post error slowing (**C**). See text for details.

Concerning Go/No-go task results, we observed significant main effect of committed errors (*F(1*.*62*, *55*.*19)* = *26*.*42*, *p* < *0*.*001*) and reaction times between conditions *(F(1*.*71*, *57*.*97)* = *11*.*91*, *p* < *0*.*001)*. Post-hoc t-tests with Bonferroni correction revealed that subjects committed fewer errors (ERRORS_PUN vs REW_ *t(35)* = −*2*,*71*, *p* < *0*.*05*, ERRORS_PUN vs NEU_ *t(35)* = −*5*,*87*, *p* < *0*.*001*) and had the slowest reaction times (RT_PUN vs REW_ *t(35)* = *4*,*86*, *p* < *0*.*001*, RT_PUN vs NEU_ *t*(35) = 3,42, p < 0.01) in the PUN condition. Subjects committed fewer errors in the REW condition than in the NEU condition (*t(35)* = −*3*,*95*, *p* < *0*.*001*). Between group comparisons revealed a trend in procrastinators to react faster (*F(1*, *34)* = *4*,*030*, *p* = *0*.*053*) and commit more errors than the LP group (*F(1*, *34)* = *2*,*887*, *p* = *0*.*098*). No differences were found in PES, as well as no significant group × condition interactions (see Fig. 3 and Table 2).

**Table 2:** Mean No-go errors, reaction time and post error slowing (PES) for the punishment, reward, and neutral condition in the Go/No-go task, for low and high procrastination group.

		Low Procrastination			High Procrastination		
		PUN	REW	NEU	PUN	REW	NEU
No-go errors	mean	23.52	26.06	31.2	31.6	34.2	40.6
[%]	SD	*14*.*36*	*15*.*85*	*18*.*02*	*14*.*32*	*16*.*86*	*16*.*36*
Reaction time	mean	396	384	384	360	353	353
[ms]	SD	66	62	58	28	32	36
PES	mean	3.8	−3.5	8.1	0.6	−1.6	10
[ms]	SD	*27*.*1*	32.*8*	*30*.*4*	*32*	*43*.*6*	40

### fMRI – event related analysis results

Brain activity related to correctly inhibited No-go trials in NEU condition revealed typical patterns in both groups (see Fig. S1; Table S1 for details) showing i.a. bilateral putamens and right DLPFC (see^46,47^ for the metaanalyses). Between-group contrasts revealed no significant clusters.

**Figure 4:**
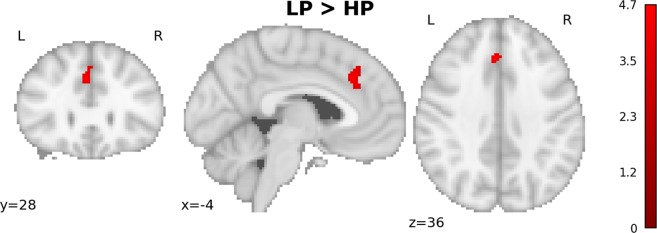
Difference in error related activity in the NEU condition. LP > HP contrast (SVC), reveals significantly stronger activation in the Anterior Cingulate Cortex.

Error related activity in the NEU condition in both groups showed i.a: ACC, bilateral insulae, DLPFC (right in HP, bilateral in LP; see Fig. S2 and Table S2). The activations seemed to be stronger in LP, and indeed LP > HP contrast revealed significant cluster within ACC (Fig. 4, Table 3).

**Table 3:** Between group differences (LP > HP) in error-related activity during NEU condition. p- and t-values presented were obtained with FWE correction at a voxel (peak) level within small volumes used as search spaces.

Region Label	Extent	t-value	p-value	X	Y	Z
Paracingulate Gyrus	129	4.68	0.017	−4	28	36

Similar between group differences in error-related activity were found for PUN and REW condition (not shown). No significant group x condition interactions in error processing were found.

### fMRI – block related analysis results

**Figure 5:**
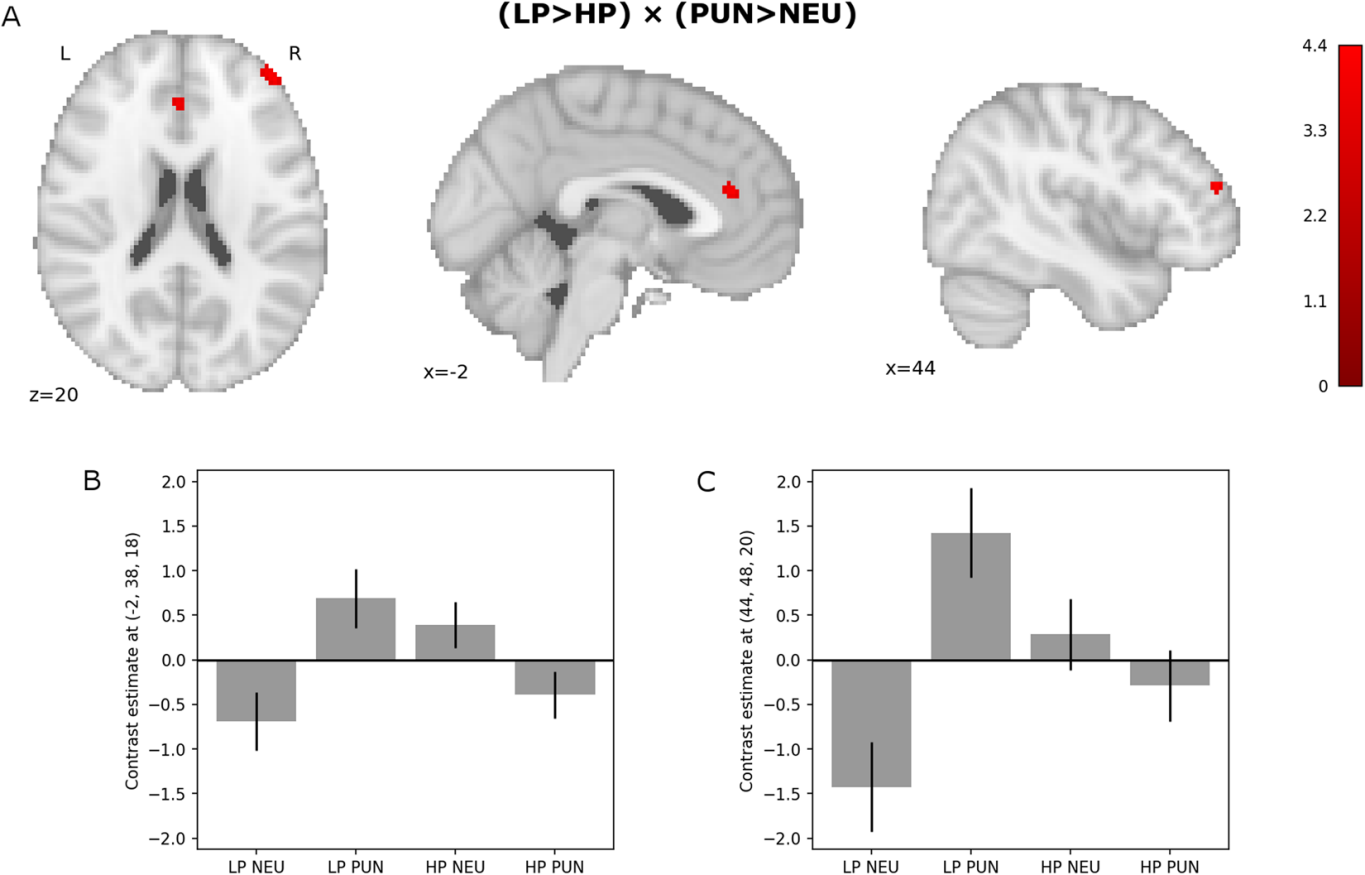
Results of (LP > HP) × (PUN > NEU) interaction (SVC). (**A**) the interaction results, (**B**,**C**) - contrast estimates and 90% C.I. in the Anterior Cingulate Cortex (**B**) and right Dorsolateral Prefrontal Cortex (**C**) presented for visualization purposes.

Group (LP > HP) by condition (PUN > NEU) interaction revealed significant difference in ACC and right DLPFC activity (Fig. 5A and Table 4).

**Table 4:** Group by condition interaction in tonic brain activity between punishment and neutral conditions. p- and t-values presented were obtained with FWE correction at a voxel (peak) level within small volumes used as search spaces.

Region Label	Extent	t-value	p-value	X	Y	Z
**(LP** > **HP)** × (**PUN** > **NEU)**						
Frontal Pole	40	4.39	0.025	44	48	20
Cingulate Gyrus Anterior Division	27	4.24	0.035	−2	38	18

Contrast estimates for peaks of the ACC and DLPFC clusters showing the group x condition interactions are presented in Fig. 5B,C respectively.

No significant interactions were found in analogous analyses for REW condition.

## Discussion

The aim of this study was to investigate the neuronal mechanisms of inhibitory control and error processing in procrastinators. Additionally, we wanted to examine the influence of negative emotions (risk of financial punishment) on the neural mechanisms of behavioral control in procrastinators. As procrastination was shown to be correlated with executive dysfunctions in the domain of inhibition^21,22^, we decided to use one of the most robust inhibitory tasks: Go/No-go. Additionally, taking into account emotion regulation difficulties and higher sensitivity to punishment, we introduced additional manipulations to the task in the form of monetary punishments and rewards.

Surprisingly, between-group differences in reaction times and task performance were only on trend level in our sample. Lack of significant differences found in performance and reaction times may be a matter of statistical power and relatively small sample sizes – the between group effect sizes for RTs and task performance were small to medium (partial eta squared of 0.11 and 0.08 respectively), suggesting that with larger groups the differences could gain significance. However it is also possible that lack of significant differences results from the fact that the Go/No-go task is relatively simple and may be not sensitive enough to detect potential between-group differences. Indeed, previously reported lower performance of procrastinators in inhibitory tasks was found with more complex and/or demanding tasks i.e. anti-saccade, Stroop^21^ and resistance to proactive interference in working memory^22^ tasks. Results of stop-signal task seem to be inconclusive (^21^ vs^22^). No differences in the Go/No-go task performance were also found in some of internet gamers studies^44,45^ although difference has been observed in one other study^66^ (for review and metaanalysis of Go/No-go task sensitivity in different psychopathology conditions see^67^).

On the neuronal level we wondered if the high procrastinators’ brain activity during correct inhibition trials would be weaker (e.g. similarly to substance use, review in^43^) or stronger (like in behavioral addictions, review in^43^) as compared to low procrastinators. Between group comparison however did not show any statistically significant regions, suggesting that inhibition deficits in procrastination, if they exist, might not be very severe.

Unlike in our previous behavioral study on a student population^38^, we did not find significant between-group differences in post-error reaction times. Lack of differences in PES, especially in the PUN condition, may be a result of slight differences in the task design. In the present study, longer intervals between No-go trials (at least 4 Go trials) were used and subjects could have realized that No-go trials were followed by Go trials. The difference between both studies may also indicate that procrastination-related results obtained from students may not necessarily apply to a general population (cf.^29^). However procrastinators showed significantly lower error related activity within the cingulate than low procrastinating subjects. Similar results were found in addictions (both behavioral and substance, metaanalysis in^43^), ADHD^68^, and criminal offenders (lower after error ACC activity in Go/No-go task was found to be a strong predictor of subsequent rearrest^69^). Similarly, in EEG studies, error-related negativity (ERN) was found to be reduced in impulsivity^70^ and several addictions (metaanalysis in^43^). This could suggest that attenuated error-related activity of ACC may be related to more general self-control problems. The obtained results may also be in line with^26^ showing lower resting-state functional connectivity (RSFC) between dorsal ACC and caudate nucleus. This was interpreted by the authors as procrastinators’ “difficulties in weighting response-outcome values and in linking behaviors to outcomes, which results in failure of performance monitoring and inhibition of counter-goal behaviors”. Impaired error processing may be the reason for persistence of procrastination, as the faulty behaviors might not be processed deeply enough to be effectively corrected for. In our study we did not find significant group (HP vs LP) x condition (NEU vs PUN) interaction in error processing, this however could be due to the GLM model used, separating context-related tonic activity from event-related activity (see Methods; cf.^39^). In previous EEG studies however, reduced error-related negativity in the punishment conditions was found in impulsive^71^ and antisocial^72^ subjects, suggesting decreased ACC activity and reduced monitoring under risk of punishment.

In the analysis of tonic context-related brain activity however, a significant group (LP > HP) by condition (PUN > NEU) interaction in the right DLPFC and ACC was found. This was driven mostly by the increase of activity in these structures during punishment condition in the LP group. Such a tonic increase in the ACC activity during punishment condition was found in very similar study on the general population^39^. The authors interpreted it as a “reflection of accentuated proactive performance monitoring during the punishment condition when an error might result in a financial loss”^39^. Our results suggest that such monitoring is not intensified in the high procrastinating group. Analogously the increase in right DLPFC activity related to negative context, could represent an increase of conscious control aimed at preventing or minimizing possible losses. This again seem to be present in the LP but not HP group. Surprisingly, no punishment-related increase of the DLPFC activity was found in the aforementioned study^39^ on the general population. This could be related to strong individual differences in rDLPFC activation suggested by our results (see Fig. 5C). The possible roles of DLPFCs in procrastination have already been discussed in the literature. Wu and colleagues^26^ suggested that failures in impulse control may be related to negative correlation of procrastination with RSFC between DLPFC and ventromedial PFC which was found in their study. Liu & Feng^25^ showed that procrastination correlates negatively with gray matter volume (GMV) in left DLPFC. Moreover, left DLPFC was one of the regions where GMV correlated negatively with impulsivity, suggesting again that procrastination and impulsivity might share some mechanisms which undermine self-control. Apparent lack of increase of ACC and right DLPFC activity in high procrastinators in punishment context might indicate mechanisms in which negative emotions disturb higher order control and monitoring processes.

Indeed, abundant research shows that negative affect significantly increases procrastinating behaviors which can serve as a short-term mood regulation strategy^29,73^ (review in^18^). This is in line with questionnaire results obtained in the present study. First, procrastinators are more impulsive in four out of five scales of UPPS-P including negative impulsivity, supporting previous results on the relationship between procrastination and impulsivity (e.g.^13,21,23^, review in^4^). Second, consistent with our previous behavioral study on a student population^38^, we found high procrastinators to be far more susceptible to punishment i.e. more prone to avoid a situation possibly resulting in punishment, than low procrastinators. Indeed, despite lack of correlation of procrastination with higher anxiety^20^, procrastinators, when asked the reason for their procrastination, most often indicate “fear of failure”^30,74^.

Real-life situations (more challenging than the Go/No-go task) when paired with negative emotion, can require enhanced monitoring and top-down control, otherwise such situations can lead to lower performance and in turn cause additional negative emotions like stress and/or guilt. These emotions can then further affect self-control. This seems to be true in the case of procrastinators who often fail in self-control or “give in to feel good” in the face of emotional distress^18,35^, what may lead to further stress^75^. The mechanisms by which negative emotions undermine self-control in procrastinators requires further studies.

### Limitations

We did not measure negative emotions nor motivation induced by the punishment context. Thus we cannot separate motivational and emotional components of the punishment context. Nevertheless, we believe that the punishment context made the task more aversive and thus evoked emotional distress, which seems to be crucial in undermining self-control in procrastination^18^.

### Concluding Remarks

Our results suggest that procrastinators have impaired error processing mechanisms. It has already been suggested that self-control training can transfer between different domains (e.g.^76,77^, for review see^78^). It would be worthwhile to examine if procrastinators’ poorer performance in inhibition^21,22^ and error processing (^38^ and the present results) could be improved by impulse-control training. Moreover, procrastination appears to be related to a decreased ability to intensify self-control in more demanding situations and/or impaired coping in the context of negative situations. This suggests that interventions aiming at reducing procrastination could address emotion regulation difficulties. Indeed, it was already shown that procrastinators can benefit from emotion regulation trainings^30^. At the moment however, cognitive-behavioral therapy (CBT), seems to give the best results in treating procrastination (metaanalyses in^10,11^). In the future, more effective, tailored interventions will potentially combine impulse-control and emotion-regulation training with CBT.

## Supplementary information

Supplementary Materials

## Data Availability

The datasets generated and/or analyzed during the current study are available from the corresponding author upon request.
