# Supplementary material for: Attenuated brain activity during error processing and punishment anticipation in procrastination – a monetary Go/No-go fMRI study
Source: Sci Rep. 2019 Aug 7;9:11492. doi: 10.1038/s41598-019-48008-4 (PMC6685938; doi:10.1038/s41598-019-48008-4)
Supplement: Supplementary file 1 — Supplementary Materials [file 41598_2019_48008_MOESM1_ESM.pdf]

Supplementary Figures and Tables to:

Attenuated brain activity during error processing and punishment anticipation in procrastination – a monetary Go/No-go fMRI study

Marek Wypych*[1], Jarosław M. Michałowski[2], Dawid Droździel[1], Magda Borczykowska[3], Michał Szczepanik[1], Artur Marchewka[1]

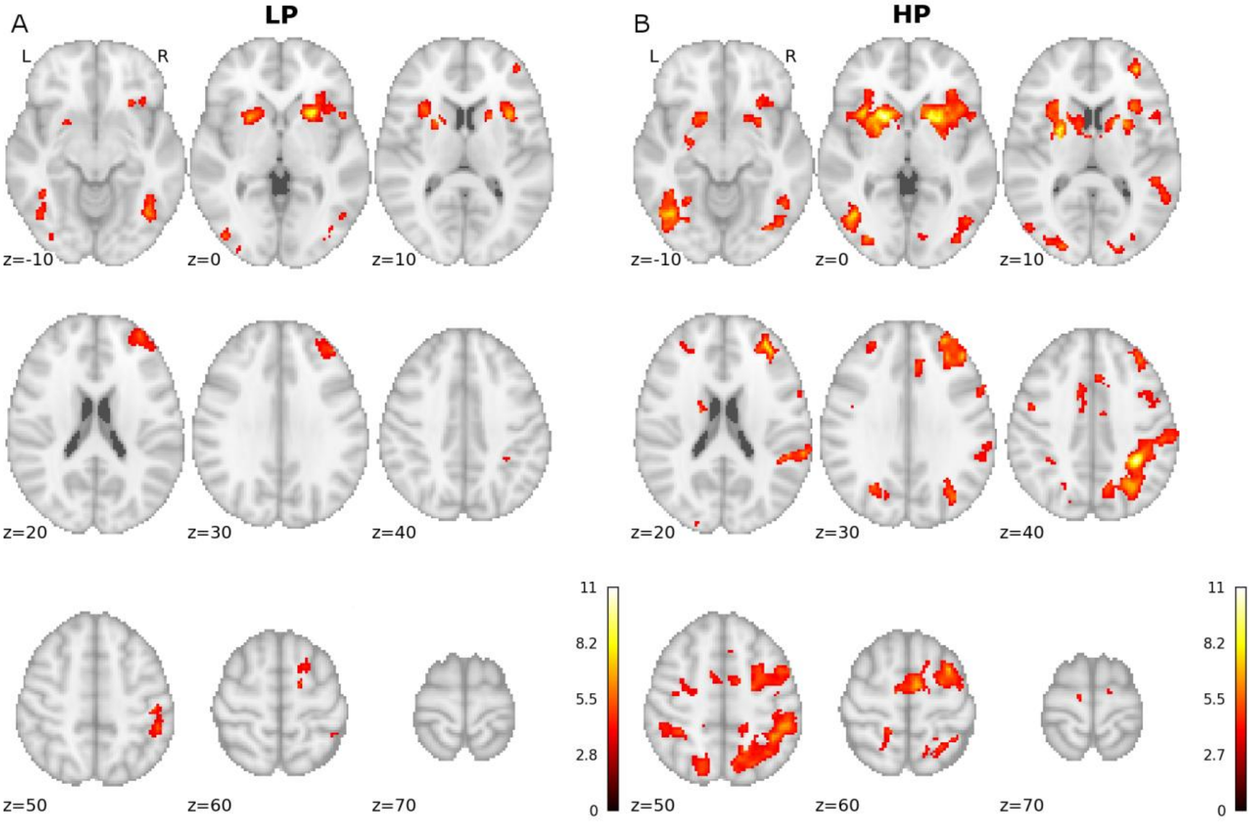

Figure S1. Activations related to correctly inhibited No-go trials in NEU condition. Panels A and B show activations found in low (LP) and high procrastination (HP) groups, respectively ($p<0.05$ FWEc). No significant between-group differences were found.

Table S1.

Activation during correct inhibitions to No-go trials for LP and HP groups separately. Within-group analysis was based on one-sample t-tests. Cluster size correction for family-wise error was used to account for multiple comparisons.

**LP Group**	**Thresholding**	t > 3.65; p < 0.001; df = 17; minimum extent = 105

Contrast Name				MNI Coordinates		
	Region Label	Extent	t-value	x	y	z
Positive	Right Putamen	836	9.153	24	14	0
	Frontal Operculum Cortex		7.337	36	14	8
	Insular Cortex		7.182	36	22	-6
	Temporal Occipital Fusiform Cortex	316	8.651	38	-54	-12
	Left Putamen	536	7.060	-24	10	0
	Left Putamen		6.945	-24	6	8
	Frontal Operculum Cortex		6.071	-30	16	12
	Temporal Occipital Fusiform Cortex	121	5.815	-40	-58	-14
	Lateral Occipital Cortex, inferior division		4.865	-44	-66	-10
	Postcentral Gyrus	328	5.794	46	-24	50
	Supramarginal Gyrus, posterior division		5.415	48	-38	50
	Superior Parietal Lobule		4.787	40	-46	56
	Lateral Occipital Cortex, inferior division	107	5.593	-44	-82	0
	Occipital Pole		4.853	-30	-92	4
	Lateral Occipital Cortex, inferior division		4.749	-38	-86	-8
	Frontal Pole	462	5.588	34	42	34
	Frontal Pole		5.533	34	54	20
	Frontal Pole		5.187	40	46	16
	Juxtapositional Lobule Cortex (formerly Supplementary Motor Cortex)	111	5.524	16	-4	60
	Superior Frontal Gyrus		4.312	18	18	58

**HP Group**	**Thresholding**	t > 3.65; p < 0.001; df = 17; minimum extent = 84				
Contrast Name				MNI Coordinates		
	Region Label	Extent	t-value	x	y	z
Positive	Superior Parietal Lobule	3360	10.967	34	-44	40
	Supramarginal Gyrus, posterior division		8.418	42	-44	46
	Left Putamen	1768	10.958	-18	10	2
	Left Putamen		10.619	-26	10	4
	Left Putamen		10.131	-26	-2	8
	Right Putamen	1645	10.567	26	4	6
	Right Putamen		10.213	16	12	-2
	Right Putamen		9.325	24	16	-4
	Frontal Pole	1036	9.027	32	46	18
	Frontal Pole		7.236	28	50	32
	Lateral Occipital Cortex, inferior division	1193	8.514	-42	-66	-2
	Occipital Fusiform Gyrus		8.256	-40	-64	-12
	Lateral Occipital Cortex, inferior division		7.720	-30	-88	4
	Middle Frontal Gyrus	1954	7.120	32	6	60
	Juxtapositional Lobule Cortex (formerly Supplementary Motor Cortex)		6.983	6	-6	60
	Middle Frontal Gyrus		6.418	28	0	54
	Lateral Occipital Cortex, superior division	130	6.549	-26	-66	26
	Cuneal Cortex		4.899	-18	-72	28
	Inferior Temporal Gyrus, temporooccipital part	470	6.285	48	-54	-14
	Lateral Occipital Cortex, inferior division		6.179	38	-84	2
	Postcentral Gyrus	293	6.265	-16	-42	58
	Superior Parietal Lobule		5.509	-34	-42	50
	Postcentral Gyrus		4.539	-12	-50	66
	Paracingulate Gyrus	218	6.166	4	22	36
	Paracingulate Gyrus		6.099	8	34	28
	Precentral Gyrus	189	6.011	-40	-12	44
	Precentral Gyrus		5.788	-48	-4	38

	Lateral Occipital Cortex, superior division	290	5.749	-16	-72	52
	Precuneous Cortex		4.698	-10	-76	44
	Intracalcarine Cortex	101	5.070	10	-88	6
	Intracalcarine Cortex		4.193	10	-80	0
	Frontal Pole	110	4.979	-26	46	28
	Frontal Pole		4.635	-32	46	22
	Frontal Pole		3.950	-32	50	32
**Note**	Table shows all local maxima separated by more than 8 mm. Regions were automatically labeled using the HarvardOxford-maxprob-thr0 atlas. x, y, and z =Montreal Neurological Institute (MNI) coordinates in the left-right, anterior-posterior, and inferior-superior dimensions, respectively.					

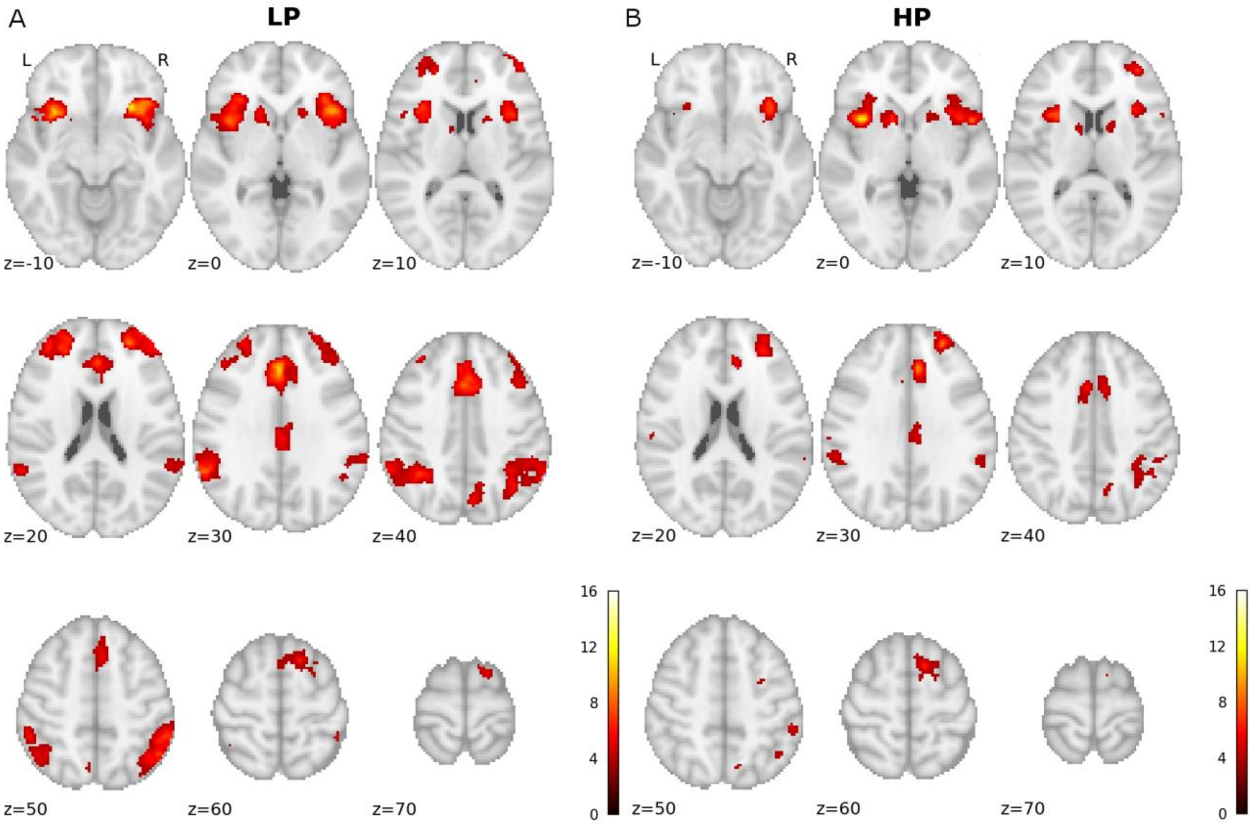

Figure S2. Error related activity in the NEU condition. Panels A and B show activations found in LP and HP groups, respectively (p<0.05 FWEc).The between-group difference is presented in the main manuscript.

Table S2.

Error related activation (reponses to No-go trials) for LP and HP groups separately. Within-group analysis was based on one-sample t-tests. Cluster size correction for family-wise error was used to account for multiple comparisons.

**LP Group**	**Thresholding**	t > 3.65; p < 0.001; df = 17; minimum extent = 99				
Contrast Name				MNI Coordinates		
	Region Label	Extent	t-value	x	y	z
Positive	Cingulate Gyrus, anterior division	2328	15.905	-2	30	26
	Paracingulate Gyrus		8.345	4	24	38
	Superior Frontal Gyrus		6.680	14	16	60
	Insular Cortex	1464	11.697	28	20	-8
	Insular Cortex		11.203	38	18	-6
	Frontal Operculum Cortex		8.565	38	16	8
	Insular Cortex	1508	11.136	-40	16	-6
	Insular Cortex		10.651	-32	18	-10
	Insular Cortex		9.542	-32	28	4
	Supramarginal Gyrus, posterior division	1696	10.254	-58	-48	26
	Angular Gyrus		6.935	-40	-56	44
	Frontal Pole	1025	8.766	-38	48	18
	Frontal Pole		7.470	-30	54	24
	Frontal Pole		7.454	-24	46	26
	Frontal Pole	1349	8.585	28	50	22
	Frontal Pole		6.564	46	48	16
	Frontal Pole		6.084	36	40	34
	Supramarginal Gyrus, posterior division	1770	7.655	52	-44	52
	Angular Gyrus		7.307	48	-56	52
	Cingulate Gyrus, posterior division	224	7.056	6	-16	28
	Cingulate Gyrus, posterior division		5.970	4	-24	28
	Left Cerebellum	99	6.445	-36	-54	-30
	Precuneous Cortex	240	5.712	6	-72	40
	Precuneous Cortex		4.745	-8	-66	44

**HP Group**	**Thresholding**	t > 3.65; p < 0.001; df = 17; minimum extent = 94				
Contrast Name				MNI Coordinates		
	Region Label	Extent	t-value	x	y	z
Positive	Insular Cortex	994	12.352	-36	12	-2
	Insular Cortex		7.934	-30	16	10
	Frontal Pole	547	9.460	26	50	28
	Frontal Pole		7.100	34	50	10
	Paracingulate Gyrus	443	9.215	6	30	30
	Paracingulate Gyrus		6.954	10	34	24
	Cingulate Gyrus, anterior division		5.526	8	14	38
	Frontal Operculum Cortex	911	8.428	48	10	0
	Frontal Orbital Cortex		7.914	36	20	-10
	Insular Cortex		6.689	32	18	2
	Superior Frontal Gyrus	320	7.662	10	10	62
	Superior Frontal Gyrus		4.436	6	18	60
	Middle Frontal Gyrus		4.372	26	-2	52
	Cingulate Gyrus, posterior division	99	7.002	4	-18	34
	Cingulate Gyrus, anterior division	153	6.300	-6	16	38
	Supramarginal Gyrus, posterior division	493	6.024	52	-40	46
	Angular Gyrus		5.742	46	-52	42
	Supramarginal Gyrus, posterior division		5.107	56	-38	36
	Parietal Operculum Cortex	166	5.269	-58	-38	26
	Supramarginal Gyrus, posterior division		4.643	-52	-44	30
	Supramarginal Gyrus, anterior division		4.477	-64	-26	28
	Precuneous Cortex	94	5.225	10	-68	46
	Right Caudate	108	4.922	14	2	14

**Note**	Table shows all local maxima separated by more than 8 mm. Regions were automatically labeled using the HarvardOxford-maxprob-thr0 atlas. x, y, and z =Montreal Neurological Institute (MNI) coordinates in the left-right, anterior-posterior, and inferior-superior dimensions, respectively.
